# Elucidating the major hidden genomic components of the A, C, and AC genomes and their influence on *Brassica* evolution

**DOI:** 10.1038/s41598-017-18048-9

**Published:** 2017-12-21

**Authors:** Sampath Perumal, Nomar Espinosa Waminal, Jonghoon Lee, Junki Lee, Beom-Soon Choi, Hyun Hee Kim, Marie-Angèle Grandbastien, Tae-Jin Yang

**Affiliations:** 1 Agriculture and Agri-Food Canada, 107 Science Place, Saskatoon, SK S7N 0X2 Canada; 2 Department of Plant Science, Plant Genomics and Breeding Institute, Research Institute of Agriculture and Life Sciences, College of Agriculture and Life Sciences, Seoul National University, Seoul, 08826 Republic of Korea; 3 Department of Life Science, Plant Biotechnology Institute, Sahmyook University, Seoul, 01795 Republic of Korea; 4 Joeun Seed, Goesan-Gun, Chungcheongbuk-Do 28051 Republic of Korea; 5 Phyzen Genomics Institute, Seongnam, 13558 Republic of Korea; 6 INRA AgroParisTech, IJPB, UMR 1318, INRA Centre de Versailles, Versailles, Cedex France; 7 Crop Biotechnology Institute/GreenBio Science and Technology, Seoul National University, Pyeongchang, 232-916 Republic of Korea

## Abstract

Decoding complete genome sequences is prerequisite for comprehensive genomics studies. However, the currently available reference genome sequences of *Brassica rapa* (A genome), *B*. *oleracea* (C) and *B*. *napus* (AC) cover 391, 540, and 850 Mbp and represent 80.6, 85.7, and 75.2% of the estimated genome size, respectively, while remained are hidden or unassembled due to highly repetitive nature of these genome components. Here, we performed the first comprehensive genome-wide analysis using low-coverage whole-genome sequences to explore the hidden genome components based on characterization of major repeat families in the *B*. *rapa* and *B*. *oleracea* genomes. Our analysis revealed 10 major repeats (MRs) including a new family comprising about 18.8, 10.8, and 11.5% of the A, C and AC genomes, respectively. Nevertheless, these 10 MRs represented less than 0.7% of each assembled reference genome. Genomic survey and molecular cytogenetic analyses validates our *insilico* analysis and also pointed to diversity, differential distribution, and evolutionary dynamics in the three *Brassica* species. Overall, our work elucidates hidden portions of three *Brassica* genomes, thus providing a resource for understanding the complete genome structures. Furthermore, we observed that asymmetrical accumulation of the major repeats might be a cause of diversification between the A and C genomes.

## Introduction

Members of the Brassicaceae represent one of the largest eudicot families, including about 338 genera and 3740 species, which have been highly diversified by complex whole genome duplication (WGD) and subsequent evolution. The *Brassica* genus includes many plants with agricultural importance as vegetables, oils, fodders, and condiments throughout the world^1^. The genetic relationship between commonly grown diploid and tetraploid *Brassica* species is described in U’s triangle model^2^. Of the six *Brassica* species in this triangle, *B*. *rapa* (AA, 2n = 2x = 20), *B*. *nigra* (BB, 2n = 2x = 20) and *B*. *oleracea* (CC, 2n = 2x = 20) are monogenomic diploids, whereas the remaining three, *B*. *juncea* (AABB, 2n = 4x = 20) *B*. *napus* (AACC, 2n = 4x = 20) and *B*. *carinata* (BBCC, 2n = 4x = 20) are allopolyploids that derived from hybridization events between different AA, BB, CC diploid species.

WGD is common in flowering plants^3^. The *Brassica* genus experienced hexaploidization approximately 16 million years ago (MYA) after diverging from the *Arabidopsis* lineage^4,5^. This lineage-specific whole-genome triplication and selection promoted diversification of the *Brassica* genome^6,7^. Consequently, *Brassica* is rich in species, genetic, and morphological diversity, for example, in terms of leafy heads, stem enlargement, flower/inflorescence modification, and or elongated roots^6^.

Repetitive elements (REs) are major players in genome reorganization and stabilization during and after WGD events that disrupt nuclear homeostasis^8^. This concept, and the high genome diversity in *Brassica*, provides a good platform with which to study and explore the evolution of polyploid genomes in relation to RE dynamics^9^. REs, which include tandem repeats (TRs) and transposable elements (TEs), constitute a major genomic fraction (up to 85%) and are responsible for genome size increases in most organisms^10^. REs influence genome architecture, diversity and evolution via homologous recombination and chromosome rearrangements such as duplication, deletion, inversion, and translocation^11^. TRs are short elements (150–400 bp), present as an array of repeats up to 1 million copies, and are localized in heterochromatic regions such as the centromeres, peri-centromeres and sub-telomeres^12–14^. Though the size of TRs are similar between taxa, the sequences may diverge – even between closely related species – because of mutation and homogenization/fixation^15^. Housekeeping nuclear ribosomal DNA (nrDNA) sequences are one of the largest tandem array repeats^16^. They are localized in the peri-centromeric regions (5S nrDNA) and nucleolar organizer regions (45S nrDNA) of most plant species, including *Brassica* ^17–19^. Growing evidence supports the importance of TRs in genome function and evolution^20–25^.

TEs are also abundant and important for genome expansion, adaptation and evolution^26–28^. Based on their transposition mechanisms, TEs are classified into two major classes: I, retrotransposons, and II, DNA transposons^29^. Retrotransposons, especially those belonging to the *Gypsy* and *Copia* families, occupy a major fraction of most plant genomes. In some cases, a major proportion of the genome is made up of only a few retrotransposon families; for example, *Del* family retrotransposons occupy about 30% of the 3.5 Gb *Panax ginseng* genome^26^. Similarly, TRs can also make up a large fraction of a genome; for instance, ~50% of the olive genome was covered by TRs^30^. Although TEs are mostly conserved in structure, significant variations have been observed, even between close species^8,31,32^. In *Brassica*, asymmetric TE amplification may be important in genetic diversity, speciation, morphological differentiation and polyploidy adaptation^6,33^.

Reference genomes are important for understanding genome structure and help to speed up functional genomics approaches to crop improvement^34^. Advances in sequencing technologies, such as next-generation sequencing (NGS), have provided insights into the structures and functions of plant genomes at an unprecedented pace^35,36^. However, achieving a pseudo-chromosome level of assembly is arduous, often because of REs. REs can, for example, hinder complete genome assembly and leave hidden gap regions, even in model organisms^37^.

The availability of whole-genome pseudo-chromosome assembly for the major *Brassica* species such as *B*. *rapa* (330 Mb out of 485 Mb), *B*. *oleracea* (385 Mb/630 Mb) and *B*. *napus* (645 Mb/1130 Mb), has enabled better understanding of the genome architectures, compositions and evolution of these species^33,38–41^. Currently, 27 Mb (5%), 155 Mb (25%), and 205 Mb (18%) of the A, C and AC genomes, respectively, are unanchored scaffolds. The available reference genome assemblies cover 80.5%, 85.7%, 75.2% of the A, C and AC genomes, respectively, leaving 19% (94 Mb), 14% (90 Mb), and 25% (280 Mb) of the genomes unassembled, mostly because of RE assembly problems^10^. Several studies have characterized and localized REs in *Brassica* genomes, including nrDNAs^16^, centromeric tandem repeats (CentB)^42^, sub-telomeric tandem repeats (STR)^17^, centromeric and peri-centromeric long terminal repeat (LTR) retrotransposons^19^, terminal-repeat retrotransposons in miniature (TRIMs), and miniature inverted-repeat transposable elements (MITEs)^43–49^.

Here, we explored the major repetitive elements of *B*. *rapa* (A genome), *B*. *oleracea* (C genome) and *B*. *napus* (AC genome) collectively using low-coverage, whole genome sequence (WGS) reads, termed the dnaLCW-RE approach. We characterized 10 major repeats including a new repeat – and inspected their genomic abundance, diversity, and distribution. This study provides insights into the interspecific and intraspecific diversity and evolution of the major *Brassica* repeats that form the previously hidden components of the *Brassica* genome.

## Results

### *De novo* assembly and mapping of low-coverage, WGS identifies high copy repeats in *B*. *rapa* and *B*. *oleracea*

We previously demonstrated that *de novo* assembly using low-coverage, whole-genome sequences (the dnaLCW approach) can be used for complete and simultaneous assembly of high-copy genomes such as the chloroplast and nuclear ribosomal DNA^50^. Here, we used a similar approach, which we named dnaLCW-RE, to identify the sequences of the major high copy REs from *Brassica* plants of the A and C genomes.

Firstly, *B*. *rapa* (Br-1-1) low-coverage (2x), WGS Illumina pair-end reads were used for *de novo* assembly; 147 118 contigs were obtained with an average depth of 13x. Contigs were ordered based on read depth, and initially, the top 50 high-depth contigs were selected for further repeat analysis. Average depth of the top 50 contigs was 2 588x (1292–8064 copies on the haploid genome equivalent) with lengths ranging between 226 and 7453 bp (Supplementary Table 1). Among these 50 contigs, 47 showed similarity to known repeat families, with 33 CentB homologs, six nrDNAs, three STRs, and five transposons. The remaining three contigs were of unknown origin and were too small for further analysis. A total of eight repeat families were characterized, including centromeric tandem repeats of *B*. *rapa* (CentBr1 and CentBr2), sub-telomeric tandem repeats (STRa and STRb), nuclear ribosomal DNA units (5S nrDNA and 45S nrDNA), centromere-specific *Brassica* retrotransposons (CRB), and peri-centromeric *B*. *rapa* retrotransposons (PCRBr1a) (Supplementary Table 2).

*De novo* assembly of *B*. *oleracea* Bol-1-1 WGS (2x haploid genome equivalents) generated 260 198 contigs. The top 50 high-depth contig lengths ranged between 200 and 2103 bp, and read depth ranged between 139 and 13 366 copies. The average contig length of *B*. *oleracea* was much larger than that of *B*. *rapa*, and the contigs were annotated based on a sequence similarity searches against the Repbase and National Center for Biotechnology Information (NCBI) databases (Supplementary Table 3). Twelve contigs represented slightly different forms of *B*. *oleracea* centromeric tandem repeats (CentBo), 9 sub-telomeric repeats (BoSTR), 8 nrDNAs, and 14 known TEs. The remaining 7 contigs were unknown repeats. Deep investigation and grouping of these major contigs based on sequence similarity led to the identification of 10 major repeat families, including nine well-known repeats and a new *B*. *oleracea*-specific *Copia* retrotransposon (BoCop-1) (Supplementary Table 4).

In addition, we also applied RepeatExplorer method to characterize major repeats using the same WGS reads. RepeatExplorer based analysis revealed 90, 107, and 284 clusters with the genome occupancy of 46%, 39% and 51% in A, C and AC genome, respectively (Supplementary Tables 5,6,7 and 8). Comparative analysis revealed that all of these RepeatExplorer clusters belonged to the 10 MRs identified through dnaLCW-RE. Moreover, those clusters containing 10 MRs occupied 21.8%, 14.6% and 12.4% of the A, C and AC genome, respectively, which is similar but slightly higher than dnaLCW-RE analysis (Supplementary Table 9). In addition, this analysis also provides information for repeats other than 10 MRs contributing significant fraction of the three *Brassica* genome.

### Characterization of a new LTR-retrotransposon family in the *B*. *oleracea* genome

**Figure 1:**
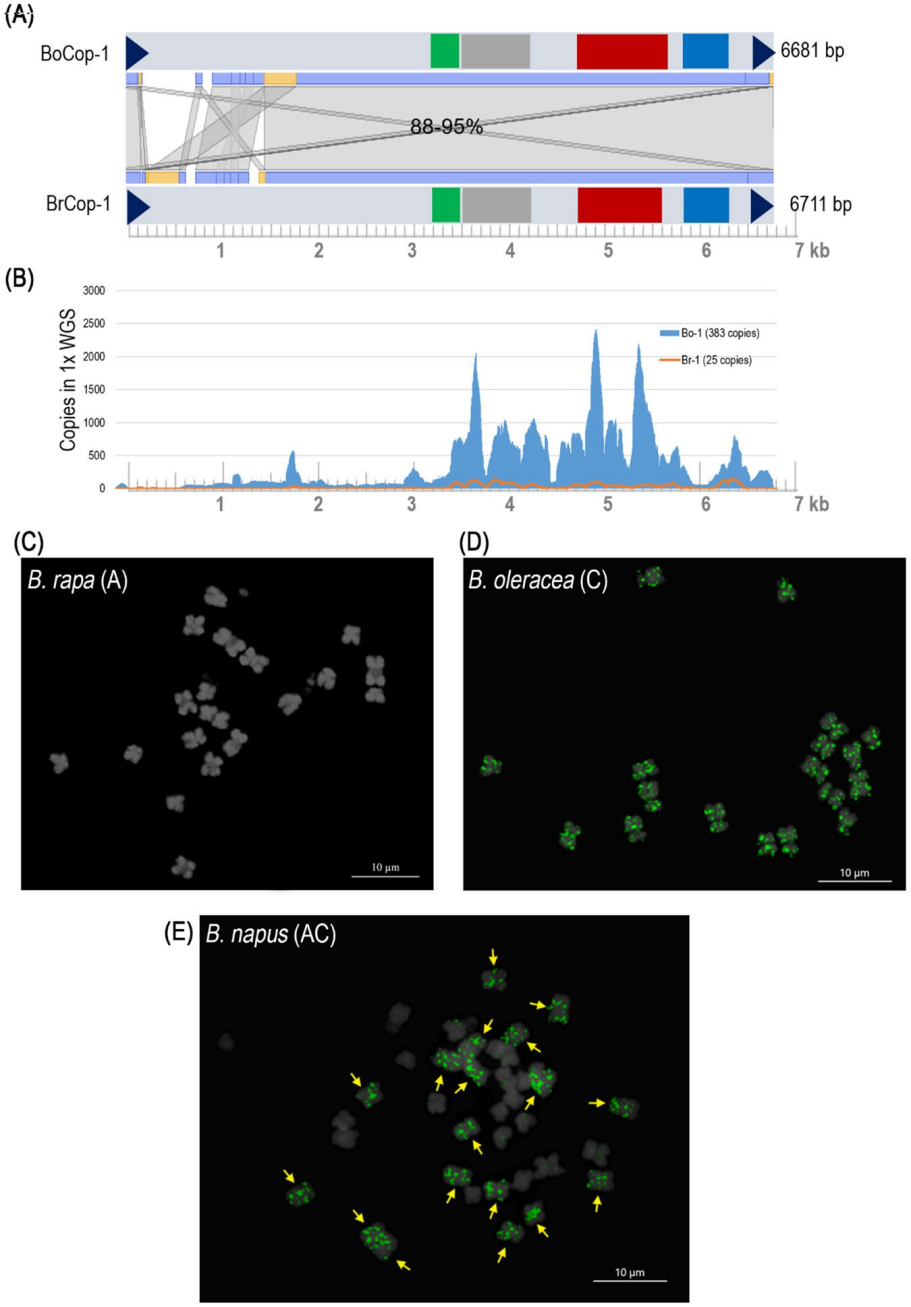
Structure and syntenic analysis of the new long terminal repeat (LTR) retrotranposon. (**A)** Micro-syntenic comparison of *B*. *oleracea*-specific LTR-RT (BoCop-1) with its ortholog (BrCop-1) from *B*. *rapa*. Arrows denote the LTR; Green, red and blue box denotes the functional domain, GAG, reverse transcriptase and integrase, respectively. Value for similarity with homologous regions is represented as a percentage. **(B)** Read mapping and copy number estimation in *B*. *oleracea* and *B*. *napus*. FISH analysis of BoCop1 in *B*. *rapa* **(C)** *B*. *oleracea* **(D)** and *B*. *napus* **(E)** genomes. Arrows indicate C sub-genomes in the *B*. *napus*-genome.

Nine of ten REs identified using the dnaLCW-RE approach were similar to those previously reported in the *Brassica* genome (Table 1). However, this approach also revealed a new, highly abundant, *B*. *oleracea*-specific LTR retrotransposon. Based on in-depth analysis of unclassified contigs from C (Fig. 1), this was characterized as a Ty1/*Copia* type (BoCop-1). Among the top 50 *B*. *oleracea* contigs, two unclassified contigs (numbers 12 and 29) were expected to represent 1423x per genome (Supplementary Table 3). A Repbase search revealed that both contigs were similar to *Copia*-type LTR retrotransposons. Contig lengths were extended by manual read walking to obtain the complete LTR retrotransposon structure. Annotation of the extended contig revealed signature structures of LTR retrotransposons, such as target site duplication, terminal repeats, and a functional coding domain. Likewise, unclassified *B*. *oleracea* contigs led to the identification of *Copia*-type LTR-retrotransposon in *B*. *oleracea* (Fig. 1A). Read mapping revealed 383 and 25 copies in the A and C genomes, respectively (Fig. 1B). Karyotype analysis using the tetraploid derivative AC genome showed C genome-specific amplification (Fig. 1C).

**Table 1:** Composition of major repeats based on the reference genome assembly and 1x WGS of three *Brassica species*.

Element ID	Element size (bp)	*B*. *rapa*						*B*. *oleracea*						*B*. *napus*						Homologous element
		Reference genome (391 Mb)			1x wgs (485 Mb)			Reference genome (540 Mb)			1x wgs (630 Mb)			Reference genome (850 Mb)			1x wgs (1130 Mb)			
		GR-R (n)	GR-R (Kb)	GP-R (%)	GR-W (n)	GR-W (Kb)	GP-W (%)	GR-R (n)	GR-R (Kb)	GP-R (%)	GR-W (n)	GR-W (Kb)	GP-W (%)	GR-R (n)	GR-R (Kb)	GP-R (%)	GR-W (n)	GR-W (Kb)	GP-W (%)	
CentB1	176	1,632	283	0.07	179,807	31,646	6.5	1,203	196	0.03	114,077	20,192	3.2	336	56	0	228,031	40,362	2.3	Lim *et al*. 2005
CentB2	176	1,182	204	0.05	36,765	6,470	1.3	1,924	317	0.05	89,827	15,899	2.5	518	85	0.001	51,093	9,043	0.5	Lim *et al*. 2005
5 S nrDNA	501	75	37	0.01	5,096	2,553	0.5	143	70	0.01	1,286	647	0.1	45	22	0	5,147	2,579	0.3	Waminal *et al*. 2015
45 S nrDNA	7,456	5	32	0.01	4,008	29,883	6.2	1	5	0	1,072	8,136	1.3	—	—	0	4,089	30,485	5	Waminal *et al*. 2015
STR-Br^a^	352	2,155	735	0.19	13,296	4,685	1	1,511	477	0.08	3,829	1,354	0.2	1,517	509	0.005	20,349	7,122	0.1	Koo *et al*. 2011
STR-Bo^b^	352	1,376	466	0.12	738	260	0.1	5,186	1,735	0.27	21,067	7,394	1.2	4,632	1,569	0.014	23,142	8,123	0.1	Koo *et al*. 2011
CRB	5,908	2	11.4	0.00	633	3,738	0.8	2	12	0	486	2,995	0.5	—	—	0	1,168	6,902	0.9	Lim *et al*. 2007
PCRBr1a	8,395	1	7.8	0.00	1,268	10,441	2.1	1	8	0	86	711	0.1	1	8	0	960.4	8,217	1.2	Lim *et al*. 2007
Cop-1	6,711	1	5.2	0.00	34	229	0.1	15	88	0.01	298	1,988	0.3	1	6	0	284.4	1,910	0.2	This study
CACTA	7,675	1	7.6	0.00	143	1101	0.2	1	9	0	956	8,987	1.4	1	8	0	1,266	9,713	0.9	Alix et al. 2008
Total		6,430	1,788.3	0.63	241,788	91,006.8	18.76	9,987	2,916.40	0.463	232,984	68,303	10.84	7,051	2,262.2	0.02	335,529	124,455	11.52	

GR-R (n): Number of repeat units represented in reference genome sequences; GR-R (kb): Total length of repeat units represented in reference genome sequences (kbp); GR-W (n): Number of repeat units represented in WGS; GR-W (kb): Total length of repeat units represented in WGS (kbp); GP-R: Proportion of the genome in reference genome sequence; GP-W: Proportion of the genome in WGS; Kb: amounts in kbp. ^a^ Based on two STRs, (STRa and STRb) of *B*. *rapa* (Br); ^b^Based on two STRs, (STRa and STRb) of *B*. *oleracea* (Bo).

### Estimation of copy numbers and genome proportions for the 10 major repeats in A, C and AC genomes

**Figure 2:**
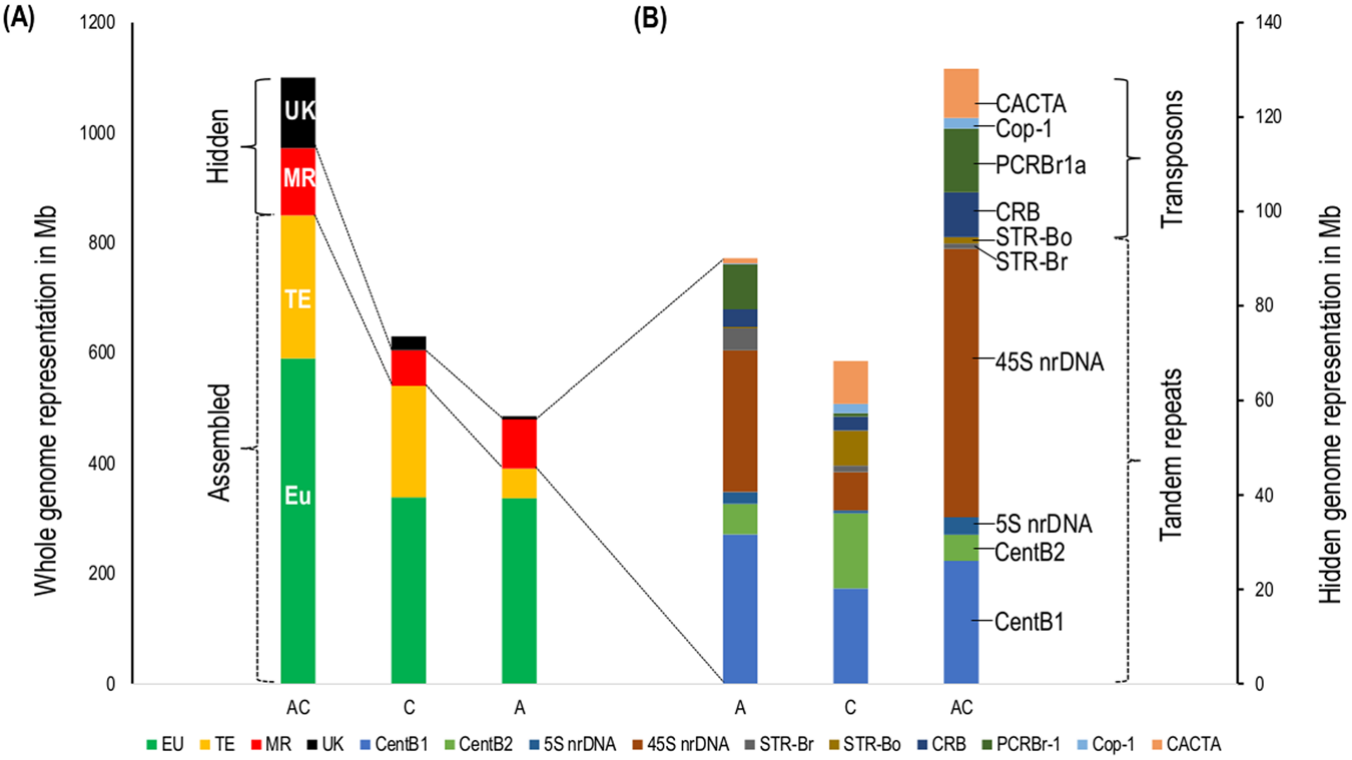
Classification of genome components based on current assembly, and elucidation of hidden genome components in three *Brassica* genomes. (**A**) The assembled reference genome was classified based on genome components such as non-repetitive euchromatic regions (EU) and repetitive TE portions (TE) from genome annotation and the hidden genome. The hidden genome is the proportion of unassembled genome. Here, red indicates the proportion of the genome occupied by 10 major repeats (MR), and gray indicates the unknown genome component (UK). (**B**) Representation of 10 MR families in the hidden genome. MR was subdivided into 10 repeat families: CentB1, CentB2, 5 S nrDNA, 45 S nrDNA, STR-Br, STR-Bo, PCRBr1a, Cop-1, CRB, and CACTA (ordered bottom to top).

The relative genomic abundance of the 10 major repeat families was quantified in the reference genome, and also in the WGSs of 64 accessions belonging to the A, C and AC genomes (Supplementary Tables 1,2,4 and 10). Comparative analysis revealed that about 19%, 11% and 12% of the A, C and AC genomes, respectively, was occupied by these 10 REs, while <0.7% was found in the reference genomes (Fig. 2).

**Figure 3:**
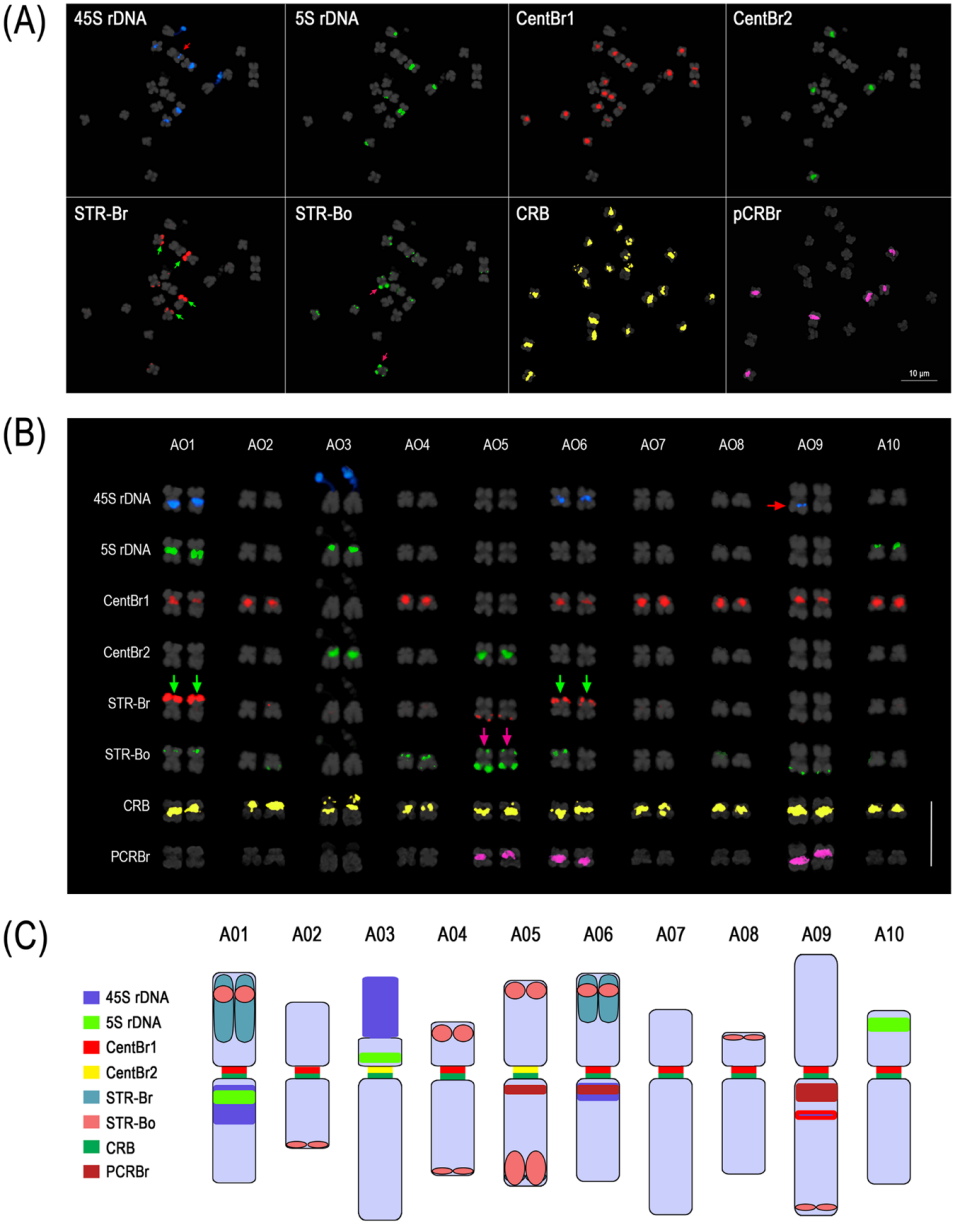
Physical mapping of major repeats in *Brassica rapa* through FISH analysis. **(A)** FISH analysis of major *Brassica* repeats. Red, green and pink arrows indicate a minor hemizygous 45 S rDNA locus, major STR-Br and STR-Bo signals, respectively. **(B)** Karyogram of *B*. *rapa* based on the distribution of major DNA tandem repeats. Green and pink arrows indicate chromosomes with major STR-Br and STR-Bo signals, respectively, which amounted to three major signals, and red arrow indicates a hemizygous 45 S rDNA. Bar = 10 µm. **(C)** Karyotype idiogram of *B*. *rapa*. 45 S rDNA with red border indicates hemizygous locus.

In the A genome, 18.8% of the genomes derived from 11 accessions, including six different subspecies, was occupied by these 10 major repeat families, while these repeats were present in less than 1% of the reference assembly (Supplementary Table 2). Total repeat length of each family varied, ranging between 0.2 Mb for BoCop-1 and 38 Mb for the two CentBr types. Of these, CentBr and 45 S nrDNA were estimated to occupy a larger fraction of the haploid genome; 7.9% and 6.2%, respectively. CentBr1 and CentBr2 were predominant components of the genome, representing 179 807 and 36 765 copies per haploid genome, respectively. Most *in silico* analyses showing the differential abundance of various repeat families were supported by analysis using fluorescence *in situ* hybridization (FISH) (Fig. 3). Different repeat families showed different chromosomal distribution patterns enabling easy identification of homologous pairs.

**Figure 4:**
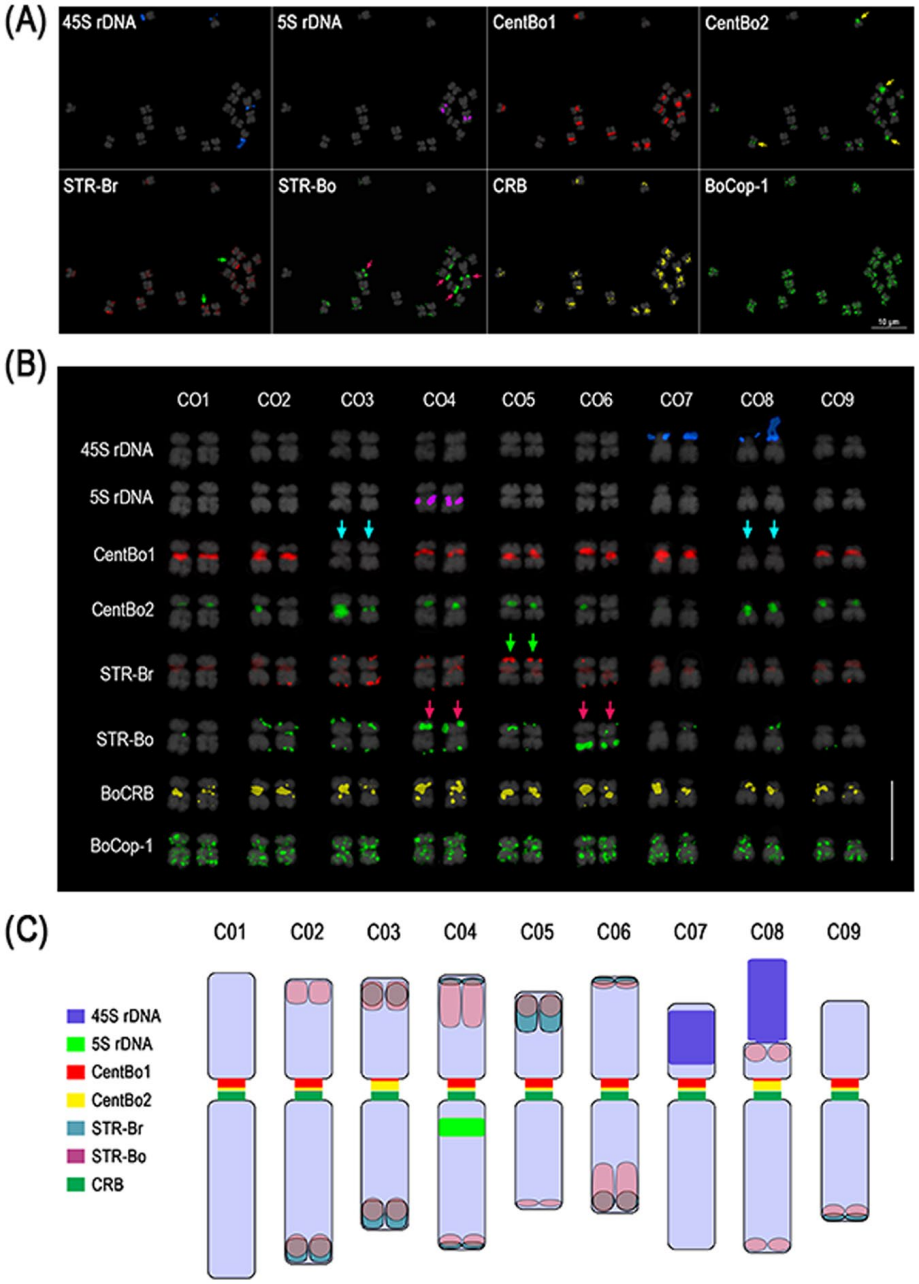
Genomic distribution of major repeats in *B*. *oleracea* through FISH analysis. **(A)** FISH analysis of major *Brassica* repeats. Green and pink arrows indicate major STR-Br and STR-Bo signals, respectively, amounting to three pairs of BoSTR signals (as observed by Koo *et al*., 2011.) **(B)** Karyogram of *B*. *oleracea* based on the distribution of major DNA tandem repeats. Blue-green arrows indicate chromosomes with very weak CentBo1 but strong CentBo2 signals. CentBo1 and CentBo2 are colocalized on most other chromosomes. Green and pink arrows indicate major signals of STR-Br and STR-Bo, respectively, which amounted to three major signals. Bar = 10 µm. **(C)** Karyotype idiogram of *B*. *oleracea*. Centromeric bars represent arbitrary signal hybridization intensities.

Analysis of 44 *B*. *oleracea* accessions – including eight different morphotypes – to interpret the repeat contribution of the genome, revealed that 10.8% of the C genome was made up of the 10 major repeat elements. However, the *B*. *oleracea* reference genome sequence contained only 2.9 Mbp of the 10 major repeats (Supplementary Table 4). Our read mapping-based calculation revealed that the major repeat proportion of the genome was 68.3 Mbp, ranging between 0.6 Mb and 20 Mb for each RE. Like *B*. *rapa*, CentBo1 and CentBo2 were present in high copy numbers, and occupied large proportions of the genome: 114 077 (3.2%) and 89 827 (2.5%), respectively. *De novo* analyses were supported by FISH using repeat-specific probes (Fig. 4). Furthermore, divergence time analysis of each high copy TR family, which revealed elements of recent and ancient origin, indicated that *B*. *rapa* had more recent amplification than *B*. *oleracea* in the span of up to 14 mya (Supplementary Figure 1).

**Figure 5:**
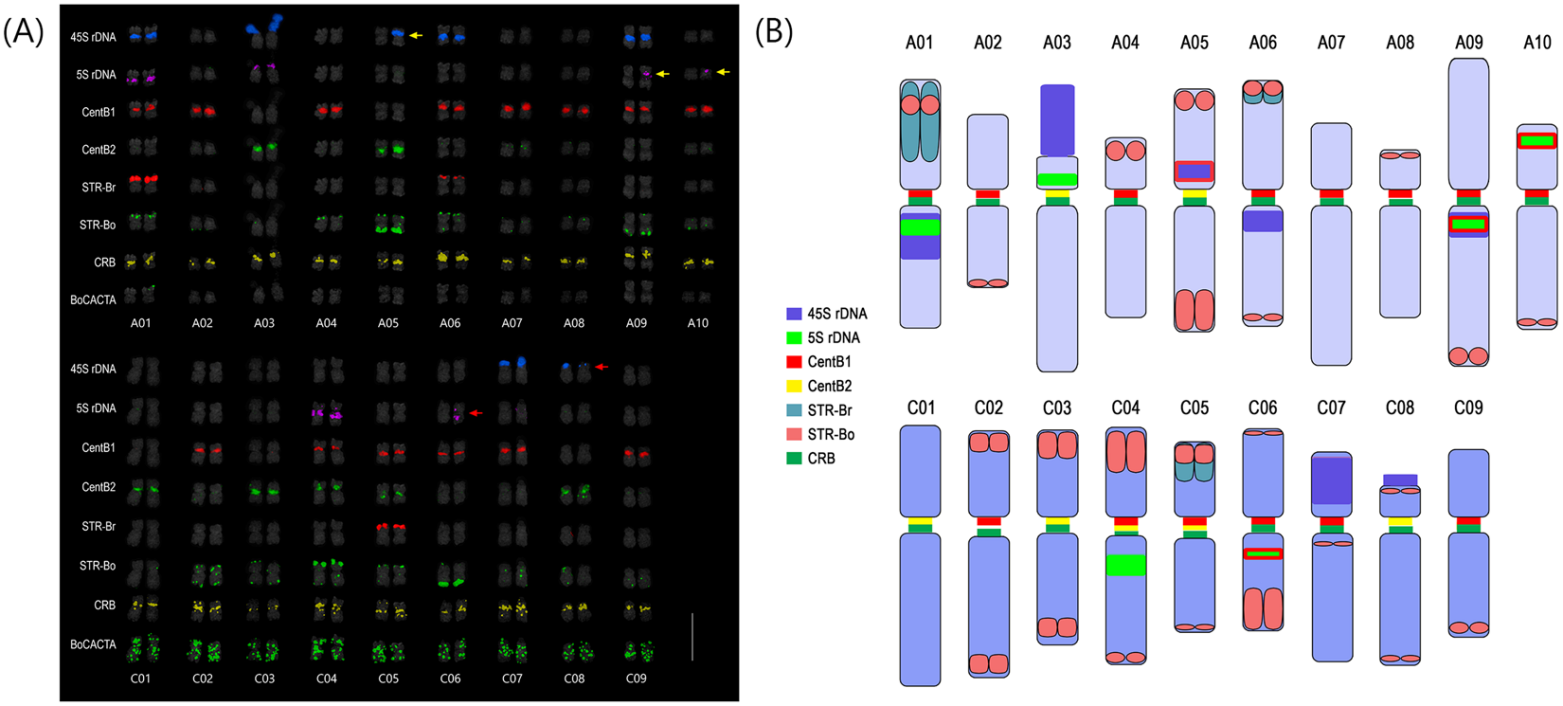
Genomic distribution of major repeats in *Brassica napus*. (**A)** Karyogram of *B*. *napus* based on the distribution of major DNA tandem repeats. Yellow and red arrows indicate major chromosomal rearrangements within the A_n_ and C_n_ genomes, respectively. Note that the C genome chromosomes, although fewer in number, are generally larger than those of the A genome, reflecting the genomic differences between the two diploid species. BoCRB is seen in all chromosomes, while BoCACTA elements are specific to C. Bar = 10 µm. **(B)** Karyotype idiogram of *B*. *napus*. rDNA with red border represents hemizygous loci, most likely from homeologous unequal crossover. Darker chromosomes of the C genome indicate the preferential hybridization of the BoCACTA transposon.

Estimating the 10 major repeats for the allotetraploid *B*. *napus* showed that they made up a significant fraction (11.5%) of the genome, albeit a much lower percentage (0.02%) represented in the genome assembly (Supplementary Tables 2 and 10). Of the 10 repeats screened in the genome, 4 088 copies of 45 S nrDNA and 228 030 copies of CentBnp1 were found, representing 5% and 2.3% of the genome, respectively. Furthermore, 45 S nrDNA contributed the highest proportion, covering 30 Mb of the haploid genome. Compared with its parental genome, *B*. *napus* had relative low amounts of major repeats from the ancestral A and C genomes, although the overall composition of repeats was slightly reduced to around 2.6%. FISH analysis based on repeat-specific probes showed the relative abundance of each repeat family for rDNA, CentBnp, STR and CRB which is well agreement with quantification based on dnaLCW-RE (Fig. 5). Moreover FISH has also approved the C sub-genome specific distribution of CACTA in *B*. *napus* genome.

### Karyotype analysis-based genomic distribution and proportion of REs

Five-color FISH analysis revealed unique patterns of repeat distributions for most of the REs in three *Brassica* genomes (Figs 3,4 and 5). For example, centromere-specific localization was observed for CRB, and CentB1 and CentB2 family repeats. CRB signals were observed in all the chromosomes of the A and C genomes. However, different FISH signal distribution patterns were observed for CentBo/Br1 and CentBo/Br2 in both genomes. CentBr1 was distinctly localized to 8 out of 10 chromosomes of *B*. *rapa*, and the remaining chromosomes (A02 and A04) were occupied by CentBr2 (Fig. 4). Unlike those observed in *B*. *rapa*, CentBo1 and CentBo2 were intermingled to different degrees in all *B*. *oleracea* chromosomes. CRB remained in all centromeres of the *B*. *napus* A_n_C_n_ chromosomes. In A_n_ chromosomes, CentBnp1 retained the pattern of CentBr1 distribution seen in A_r_ chromosomes, but CentBnp2 had a rearranged pattern. Chromosomes 2, 6, 7, and 9 had exclusively CentBnp1 signals, chromosomes 1, 3, and 8 had exclusively CentBnp2 signals, and chromosomes 4 and 5 had colocalized CentBnp signals (Fig. 5).

Unlike the centromeric tandem repeats, STRs preferentially accumulated into the sub-telomeric regions of some chromosomes. *B*. *rapa* STRs were observed in only a few chromosomes: BrSTRa was in three chromosomes, and BrSTRb was in seven chromosomes. STR-Bo repeats were present in the sub-telomeric regions of most chromosomes, although with different intensities. Patterns from the A_r_ and C_o_ genomes were retained in the A_n_C_n_ genome. In addition to chromosome-specific distribution, genome-specific amplification was observed for BoCop-1. Like BoCACTA, BoCop-1 was specific to the C genome and was widely distributed in *B*. *oleracea* chromosomes (Fig. 1).

## Discussion

### dnaLCW-RE is a useful tool to characterize the hidden components of *Brassica* and other genomes

High-throughput NGS technologies enable assembly of the genomes of many important crops at unprecedented pace and accuracy; consequently, this advance makes comparative studies and many downstream analyses possible^34,51–56^. Complete assembly of plant genomes is hampered by the complex genome structure caused by different REs^37^. Genome-wide exploration of the repetitive parts of the genome will us help to understand complete genome structures and compositions^18,57–60^.

Here, we performed the first comprehensive genome-wide analysis to identify major repeat families in the *B*. *rapa*, and *B*. *oleracea*, genomes using the dnaLCW-RE approach, which makes use of low-coverage (2x coverage) WGS. We found 10 major repeats in both the A and C genomes, including three new repeat families. Among the 10 MRs, six were common to both the *B*. *rapa* and *B*. *oleracea* genomes and four elements were specific to one or the other of the genomes, e.g., STR-Br, and PCRBr were abundant in *B rapa* whereas Cop-1 and CACTA were abundant in *B*. *oleracea*. *In silico* analysis estimated these 10 major repeat families to occupy about 19%, 11% and 12% of the A, C, and AC genomes, respectively, reflecting 48 and 76% of the proportions of the hidden genomes of A and C genomes, respectively. Tandem repeats (TRs), such as CenB, STRs, and 45 S rDNA, occupied greater portions of the *B*. *rapa* genome than the *B*. *oleracea* genome. TRs present in highly condensed arrays are difficult to assemble, thus explaining why large fractions of the *B*. *rapa* reference genome sequence have remained hidden. By contrast, TEs were amplified in the hidden genome of *B*. *oleracea* compared to that of *B*. *rapa*, although many more TEs were included in the assembled *B*. *oleracea* reference genome sequence than that of *B*. *rapa* (Fig. 2).

RepeatExplorer based repeat characterization provided wealth of support for dnaLCW-RE approach based major repeat characterization. All the 10 MRs were able to find in the RepeatExplorer output, though there was a slight difference in the estimation of genome proportions. Both approaches were significantly good at capturing complete unit of the short tandem repeats especially CentB and STR. However, RepeatExplorer produced more number of repeats in less number of contigs which shows the advantage over dnaLCW-RE approach for characterizing other repeats. Since our analysis is based on a low-coverage and *de novo* assembly approach, it is possible that some major REs have been missed, and more repeats may be identified with increased depth of contig analysis. Combining both tool will provide good opportunity to understand the complex heterochromatin structure in plant genome.

Cytogenetic analysis of these MRs revealed the genomic distribution, diversity and abundance of each repeat family in the three *Brassica* genomes, which supported our *in silico* survey. Notably, we conclude that about 40% of the A, C, and AC genomes are occupied by REs, indicating that other uncharacterized REs with moderate copy numbers (e.g., DNA transposons and retrotransposons) may be found in the hidden genome and are not represented in this survey (Fig. 2). However, these uncharacterized REs may be explored with the dnaLCW-RE method if analysis is extended to unannotated *de novo* assembled contigs or combination with RepeatExplorer approach. The hidden portion of each genome is expected to be larger than what was captured in this survey.

Most repeat families are similar to those previously reported in the *Brassica* genome, which was characterized by multiple, independent research groups (Table 1)^19,42^. Nevertheless, our approach was able to identify those MRs in a single study. Furthermore, our approach provided information about new RE in *B*. *oleracea* (BoCop-1), which will fuel future studies on genome diversification and evolution in the *Brassicaceae*.

### BoCop-1 is a C-genome specific LTR-retrotransposon

Over 370 copies of BoCop-1 were predicted in the C genome, but few were found in the A genome (Fig. 1). This indicates that the LTR retrotransposon has undergone C genome-specific amplification over the last 4.6 million years. FISH data showed BoCop-1 signals widely distributed in the *B*. *oleracea* genome, but absent in the *B*. *rapa* genome. A similar scenario was seen with the DNA transposon, BoCACTA. C genome-specific BoCACTA was exclusively amplified in the C genome after diversification with the A genome, and is expected to be important in C genome evolution and gene proliferation^61^. Like BoCACTA, BoCop-1 was also widely distributed throughout all C-genome chromosomes. The abundance and widespread nature of BoCop-1 makes it an excellent cytogenetic marker for identifying the C genome in tetraploids or unknown species. BoCop-1 may also be involved in *B*. *oleracea* evolution, diversification and speciation, like other TEs in other plants^61,62^.

### Comparative analysis of major repeats in 64 *Brassica* accessions reveals repeat dynamics in the interspecies and intraspceies *Brassica* genomes

**Figure 6:**
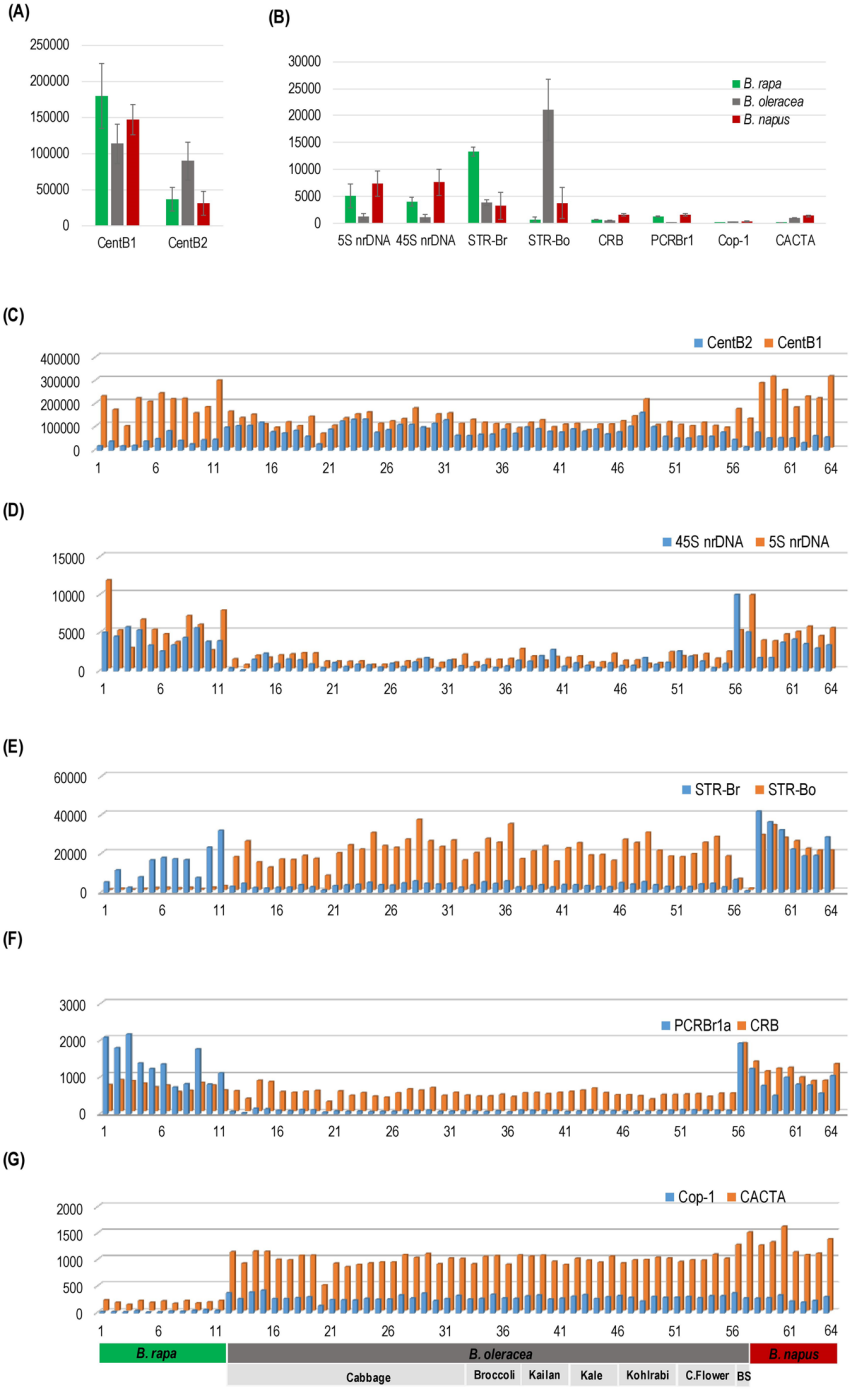
Survey of the composition of major repeats in *Brassica* genomes based on 1 × read mapping. Overall composition of repeats in these three *Brassica* genomes include centromeric tandem repeats **(**CentB-1 and CentB-2) (**A**), and 8 other repetitive elements **(B)**. Repeat abundance in 64 accessions of *B*. *oleracea* (Bo-1-44), *B*. *rapa* (Br-1-11) and *B*. *napus* (Bnp-1-9), including **(C)** CentB-1 and 2 (**D**) 5 S and 45 S ribosomal DNA (nrDNA), **(E)** STRs **(F)** centromere-specific retrotransposon of *Brassica* (CRB) and peri-centromeric retrotransposon of *B*. *rapa* (PCRBr1a), (**G**) Ty1/*Copia* retrotransposon of *Brassica* (BoCop-1), and CACTA DNA transposon of *B*. *oleracea* (BoCACTA).

Comparative analysis of REs will aid our understanding of repeat-mediated genome diversity, and genome evolution^63^. Abundance and diversity of the major repeat families were comparatively analyzed for between and within the three *Brassica* genomes based on 64 *Brassica* accessions (Fig. 6). Overall, significant copy number divergence was observed between A, C and AC genomes (Figs 2 and 6A,B). Of the three *Brassica* species surveyed, the highest proportion of the 10 MRs was found in the A genome (18.8%) compared with AC (11.5%) and C (10.8%) genomes. Large variations were observed between accessions of the different genomes, e.g., Br-8 in the A genome, and Bo-9 in the C genome represented the highest (22.56%) and lowest (4.6%) proportion of their respective genomes, demonstrating RE dynamics in these three *Brassica* genomes. Furthermore, repeat families such as CentBr1, 45 s nrDNA, STR-Br, and PCRBr were more abundant in the A genome than in the C or AC genomes, suggesting that evolution and amplification occurred after divergence from *B*. *oleracea* around 4.6 MYA (Supplementary Figure 1a).

Overall, relatively low diversity was observed within each of the three *Brassica* genomes (Fig. 6C–F). The *B*. *oleracea* genome had low divergence of the repeat fraction (3%) compared to *B*. *rapa* (>5%) and *B*. *napus* (>5%), suggesting that in the *B*. *oleracea* genome the repeat families were highly consistent and stably amplified even between different subspecies or morphotypes. This supports the idea that triplication and selection leads to genome diversification in *B*. *oleracea* ^7^. However, few families showed diversity in the A genome, especially 5S nrDNA, CentBr1 and PCRBr1a, which deviated greatly between the different cultivars analyzed. This suggests that these repeats might be involved in evolution of the C genome subspecies.

### Molecular cytogenetic analysis reveals asymmetrical evolution of major repeats of *B*. *rapa* and *B*. *oleracea*

Quantifying repeats based on FISH signals revealed that about 33%, 21%, and 30% of these respective genomes were occupied by 10 major genomic repeats, estimated to represent about 20%, 10% and 12% of the A, C, and AC genomes, respectively, based on *in silico* analysis (Fig. 2). The discrepancy between FISH and *in silico* analyses may be explained by the fact that FISH detects only two-dimensional hybridization signals from a three-dimensional chromosome structure. A wider area may also be covered by fluorescence than the actual hybridization loci. Altogether, FISH analysis provided an enhanced view of the genomic distribution and abundance of each RE. FISH-based quantification of the MRs thus enabled more accurate estimation of interspecies diversity in *Brassica* genomes, and insights into their evolution. Rapidly evolving asymmetrical amplification of MRs may promote speciation via chromosome reorganization and interruption of chromosome pairing, which can result in a reproduction barrier between organisms.

## Materials and Methods

### Plant materials

Leaf samples from 44 *B*. *oleracea* accessions were collected from two seed companies, Asia seed Co. and Joeun Seed Co., in South Korea used for resequencing. These accessions belong to eight phenotypic groups in seven subspecies (Supplementary Table 11). Total genomic DNA was extracted and purified using the modified cetyl-trimethyl ammonium bromide (CTAB) method^64^. The quantity and quality of the genomic DNA were examined using a nanodrop spectrometer.

### Genomic datasets

Approximately 5 ng total genomic DNA from each *B*. *oleracea* accession was utilized to decode genomic information using an Illumina genome analyzer (Hiseq 2000) at Macrogen, (Seoul, South Korea). Randomly sheared genomic libraries were prepared, with an insert size of 300 bp, following the 101 bp paired-end approach recommended by the manufacturer. Genome sequences of 11 *B*. *rapa* accessions belonging to eight phenotypic groups, and nine *B*. *napus*, were obtained from previous studies^40,65^. Raw reads were preprocessed using the CLC-quality trim tool to remove any remaining linker, adapter or low quality sequences. Sequences of 0.8x to 4.4x genome coverage from 64 *Brassica* accessions were utilized for further analyses (Supplementary Table 11).

### *De novo* assembly and identification of highly abundant genomic components

We previously demonstrated the use of genome-skimming approach called *de novo* assembly of low-coverage WGS (dnaLCW) to obtain complete and simultaneous assemblies of chloroplast and nuclear ribosomal DNA genomes^50^. Low-coverage WGS, approximately 2x haploid genome-equivalent of NGS reads from *B*. *rapa* and *B*. *oleracea*, were used to independently retrieve the major and most highly abundant repetitive regions using a bioinformatics pipeline called dnaLCW-RE (Supplementary Figure 2). *De novo* assemblies of 2x haploid genome-equivalent WGS *B*. *rapa* (Br-1-1) from the quality reads filtered by the CLC-quality trim tool, were then assembled using CLC genome assembler (ver. 4.06, CLC Inc, Rarhus, Denmark) with 200–500 bp autonomously controlled overlap size. Genomic abundance in terms of average read depth (RD), along with the length of the contig (LC), were identified using a CLC reference assembly approach. The top 50 contigs of greatest depth were retrieved according to highest genomic representation (RD x LC). These were then annotated by BLASTn (best hit) against the *Repbase* database^66^ and *Brassica*TED internal database, using previously reported *Brassica* REs, and Genbank. REs were classified as known repeats if contigs shared 80% similarity and 80% sequence alignment according the 80:80 rule^67^. Partial or truncated repeats containing contigs were manually analyzed and characterized with a complete structure based on reference sequence information or manual reads. Likewise, the *B*. *oleracea* (Bo-1-1) genome sequence was independently analyzed to identify the major REs using the abovementioned approach. Sequences of the individual repeat families for each species were stored in the *Brassica*TED^48^. In addition, graph-based clustering and characterization of repetitive elements by RepeatExplorer was performed using the 0.1x WGS reads from each *B*. *rapa*, *B*. *oleracea*, and *B*. *napus* genome^68^. Clustering was performed with the criteria of more than 90% sequences similarity and at least 55% sequence length cover were likely to be grouped into a single cluster. Clusters were characterized against *Repbase* database.

### Quantification of repeat proportion in the three *Brassica* genome assemblies

Whole-genome pseudo-chromosome assemblies with unanchored scaffold sequences were obtained for *B*. *rapa* (v2.1), *B*. *oleracea* (v1.0), and *B*. *napus* (v4.1) from Genbank and turned into an in-house database. Total copies of the MRs were identified based on BLASTn searches against the corresponding *Brassica* reference assembly. We followed the universal 80:80 rule for identification of members including intact and diverse members from the three reference sequences^67^. In this approach, a repeat element should have at least 80% sequence similarity and 80% sequence coverage to be considered a full-length element^44^. Members were then classified based on maximum identity, i.e., if hits were produced at the same position with more than 80% sequence similarity between them (especially for TRs)^33^. Copy numbers of the each repeats were estimated by read depth (RD) approach quantified using WGS from 64 *Brassica* accessions. RD approach has been one of the major approach for copy number estimation. The basis of RD approach is that to calculate the depth of the coverage of a genomic region is corresponds with the copy number of the region which are expected to provide relatively accurate estimation^69^. Assuming that the WGS reads used in this experiment were randomly sampled without bias, abundance was then quantified using WGS from 64 *Brassica* accessions based on the CLC-reference assembly. Paired-reads were mapped to the MRs with high threshold set at greater than 80% identity and over 50% of the read length. And the overall mean of the read depth were calculated according to the numbers of reads were mapped on to the MRs. Outputs were normalized to 1x genome coverage for *B*. *rapa*, *B*. *oleracea*, and *B*. *napus* genomes based on corresponding genome sizes. And copies of MRs were multiplied by its size to calculate the MR abundance in total genome (GA) and the genomic proportion of each MR representing total genome was calculated.

### Fluorescence *in situ* hybridization analysis

Mitotic metaphase chromosome spreads were obtained from root samples from commercial hybrid seeds *B*. *oleraceae* ssp. *capitata* ‘Sun Power’, *B*. *rapa* ssp. *pekinensis* ‘Saeronam Spring’ (Asia Seed Company, Korea) and *B*. *napus* ssp. *napus* ‘Tapidor’ (*Brassica* seedbank, Chungnam National University, South Korea) according to a previous study^47^.

Repeat-specific probes were developed based on multiple sequence alignment, and primers were designed using the NCBI primer BLAST tool (Supplementary Table 12). Repeat-specific probes were then confirmed via PCR amplification of *B*. *oleracea* and *B*. *rapa* genomic DNA. Probes were labeled by direct nick translation using the fluors mentioned in Supplementary Table 12. The hybridization solution contained 50% formamide, 2x saline-sodium citrate buffer, with or without 5 ng/µl salmon sperm DNA, 10% dextran sulfate, and 25 ng/µl of each DNA probe, adjusted to a total volume of 40 µl/slide with DNase-free and RNase-free water (Sigma, USA, #W4502). FISH experiments, including slide pre-treatment, probe hybridization and signal detection, were carried out as reported by Waminal *et al*. (2012).

## Electronic supplementary material

Supplementary Figures 1-2

Supplementary Tables 1–12
