# Supplementary material for: Elucidating the major hidden genomic components of the A, C, and AC genomes and their influence on Brassica evolution
Source: Sci Rep. 2017 Dec 21;7:17986. doi: 10.1038/s41598-017-18048-9 (PMC5740159; doi:10.1038/s41598-017-18048-9)
Supplement: Supplementary file 1 — Supplementary Figures 1-2 [file 41598_2017_18048_MOESM1_ESM.doc]

**Elucidating the major hidden genomic components of the A, C, and AC genomes and their influence on *Brassica* evolution**

Sampath Perumal^1,2^, Nomar Espinosa Waminal^2,3^, Jonghoon Lee^4^, Junki Lee^2^, Beom-Soon Choi^5^, Hyun Hee Kim^3^, Marie-Angèle Grandbastien^6^, and Tae-Jin Yang^2,7*^

**Supporting Information - Supplementary Figures 1-2**

**
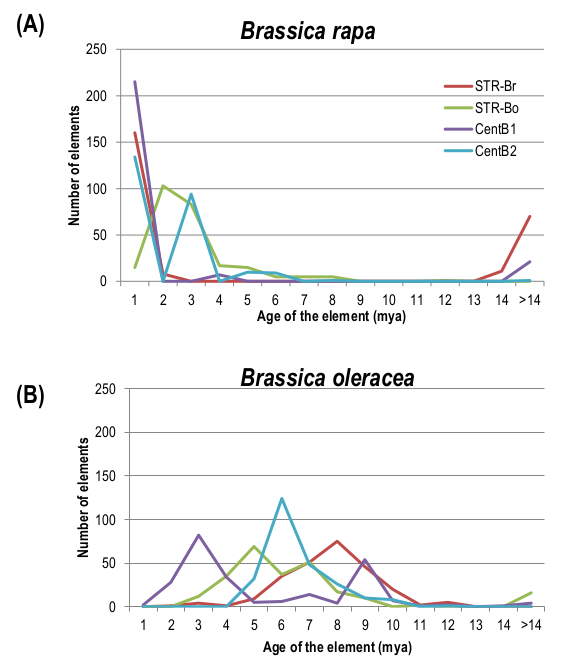
**

**Supplementary Figure 1.** Age distribution of tandem repeats (STR-Bo, STR-Br, CentB1, and CentB2) family members in the *B. rapa* (A) and *B. oleracea* (B) genomes. Base substitution rate per site (k) was estimated based on 250 intact members from four tandem repeat families by the Kimura 2-parameter distance method implemented in the MEGA 7 program for each family member against the consensus sequence from each family, followed by age estimation using the formula T = k/2r, assuming r = 1.30 × 10^−8^ ^70^.

**
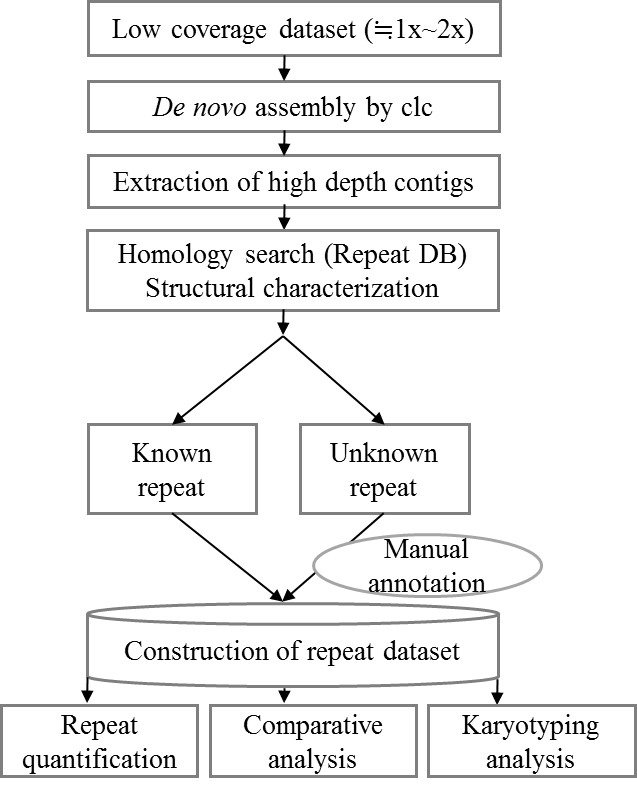
**

**Supplementary Figure 2.** Outline of the dnaLCW-RE approach, which is modified from the dnaLCW method.
